# Comparison of unipedicular and bipedicular kyphoplasty for treating acute osteoporotic vertebral compression fractures in the lower lumbar spine: a retrospective study

**DOI:** 10.1186/s12891-023-06545-0

**Published:** 2023-05-23

**Authors:** Yunfan Qian, Yimin Li, Guangjie Shen, Xiqiang Zhong, Chengxuan Tang, Shaoqi He

**Affiliations:** Department of Orthopaedic Surgery, Third Affiliated Hospital of Wenzhou Medical University, 108 WanSong Road, Ruian, Wenzhou, Zhejiang China

**Keywords:** Osteoporotic vertebral fracture, Minimally invasive surgery, Osteoporosis, Percutaneous kyphoplasty, Unipedicular, Bipedicular

## Abstract

**Background:**

Unipedicular and bipedicular approaches for percutaneous kyphoplasty are reportedly both effective in treating osteoporotic vertebral compression fractures (OVCFs). However, most studies have reported thoracolumbar fractures, with few reports describing the treatment of the lower lumbar spine. Here, we compared the clinical and radiological results of unipedicular and bipedicular approaches for percutaneous kyphoplasty for treating osteoporotic vertebral compression fractures.

**Methods:**

We retrospectively reviewed the records of 160 patients who underwent percutaneous kyphoplasty for lower lumbar (L3–L5) osteoporotic vertebral compression fractures between January 2016 and January 2020. Patient characteristics, surgical outcomes, operation time, blood loss, clinical and radiological features, and complications were compared between two groups. Cement leakage, height restoration, and cement distribution were calculated from the radiographs. Visual pain analog scale (VAS) and Oswestry Disability Index (ODI) were calculated before surgery, immediately post-surgery, and 2 years after surgery.

**Results:**

The mean age, sex, body mass index, injury time, segmental distribution, and morphological classification of fractures before surgery did not differ significantly between the groups. The results showed significant improvements in the VAS score, ODI score, and vertebral height restoration in each group (*p* < 0.05), with no significant differences between the two groups (*p* > 0.05). The mean operation time and extent of blood loss were lower in the unipedicular group than those in the bipedicular group (*p *< 0.05). Different types of bone cement leakage were observed in both groups. Leakage rate was higher in the bipedicular group than in the unipedicular group. Patients in the bipedicular group showed greater improvement in bone cement distribution than those in the unipedicular group (*p* < 0.05).

**Conclusions:**

The clinical and radiological results of unipedicular percutaneous kyphoplasty for treating osteoporotic vertebral compression fractures in the lower lumbar region were similar to those of bipedicular percutaneous kyphoplasty. However, the unipedicular approach resulted in shorter surgical time, less blood loss, and less bone cement leakage. Thus, the unipedicular approach may be preferable owing to its several advantages.

## Background

Osteoporotic vertebral compression fractures (OVCFs) have become increasingly common in older patients in recent years. Owing to a decrease in bone mass and an increase in bone fragility, even minimal trauma may be sufficient to cause fractures in older patients [1–3]. Various techniques have been developed to treat OVCFs. Among these, percutaneous balloon kyphoplasty (PKP) has been the most effective treatment for OVCFs since it was first described in 1997 [4]. PKP is a minimally invasive spinal surgery technique in which a balloon tamp is inserted into the vertebral body through the pedicle to repair the vertebral height and fix the fracture by injecting polymethyl methacrylate (PMMA) bone cement into the vertebral body [5, 6]. Currently, the standard PKP technique involves a bipedicular approach. In recent years, unipedicular approach has been advocated because of shorter operating and radiation exposure times, which can lower the risk of cement leakage and complications. Most previous studies have shown that the clinical and radiological results of unipedicular percutaneous kyphoplasty for OVCFs are similar to those of bipedicular percutaneous kyphoplasty [7–9]. However, the previous studies have mostly focused on the thoracolumbar spine, and there are few reports on the lower lumbar spine (L3–L5). Owing to the large vertebral bodies, the puncture needle does not easily reach the opposite side in the unilateral approach, and the bone cement filler cannot be evenly distributed throughout the vertebral body; therefore, most surgeons prefer the bipedicular approach. To confirm that both procedures can be successfully used in patients with lower lumbar vertebral compression fractures, we compared the clinical and radiological results of the unipedicular and bipedicular approaches for PKP to treat lower lumbar OVCFs.

## Methods

### Patients

We retrospectively analyzed patients with OVCFs of the lower lumbar spine (L3–L5) who underwent PKP between January 2016 and January 2020. The inclusion criteria were (1) age > 50 years, (2) single-level OVCFS, and (3) preoperative magnetic resonance imaging (MRI) performed to assess acute fractures, in which T2-weighted short tau inversion recovery sequence (STIR) showed obvious bone edema in the fractured vertebral body, and (4) PKP performed within 4 weeks of OVCFS occurrence, and (5) the patients received anti-osteoporosis therapy (calcium supplementation and vitamin D) and rehabilitation training postoperatively. The exclusion criteria were (1) OVCFS treated with percutaneous mesh container-plasty, (2) multiple lesions or previous compression fractures, (3) symptoms of neurological deficits, (4) severe spinal deformities or severe comorbidities (ankylosing spondylitis, multiple myeloma, tumor, thyroid or parathyroid disease, hepatic disease, and kidney disease), and (5) vertebral compression fracture due to causes other than osteoporosis. In total, 160 patients were enrolled in this study. The patients were divided into two groups according to the PKP approach: the unipedicular group (*n* = 82) and the bipedicular group (*n* = 78). The following data were collected for demographic analysis: age, sex, body mass index (BMI), injury time, segmental distribution, and morphological classification of the fractures. The fractures were classified as A (wedging), B (biconcavity), and C (crush) based on morphology, according to the EVOSG classification published in 1999 [10].

### Surgical techniques

The PKP was performed by a single surgeon. All procedures were performed under local anesthesia. The patients were placed in the prone position on four bolsters on a radiolucent operating table with the abdomen freely suspended.

**Fig. 1:**
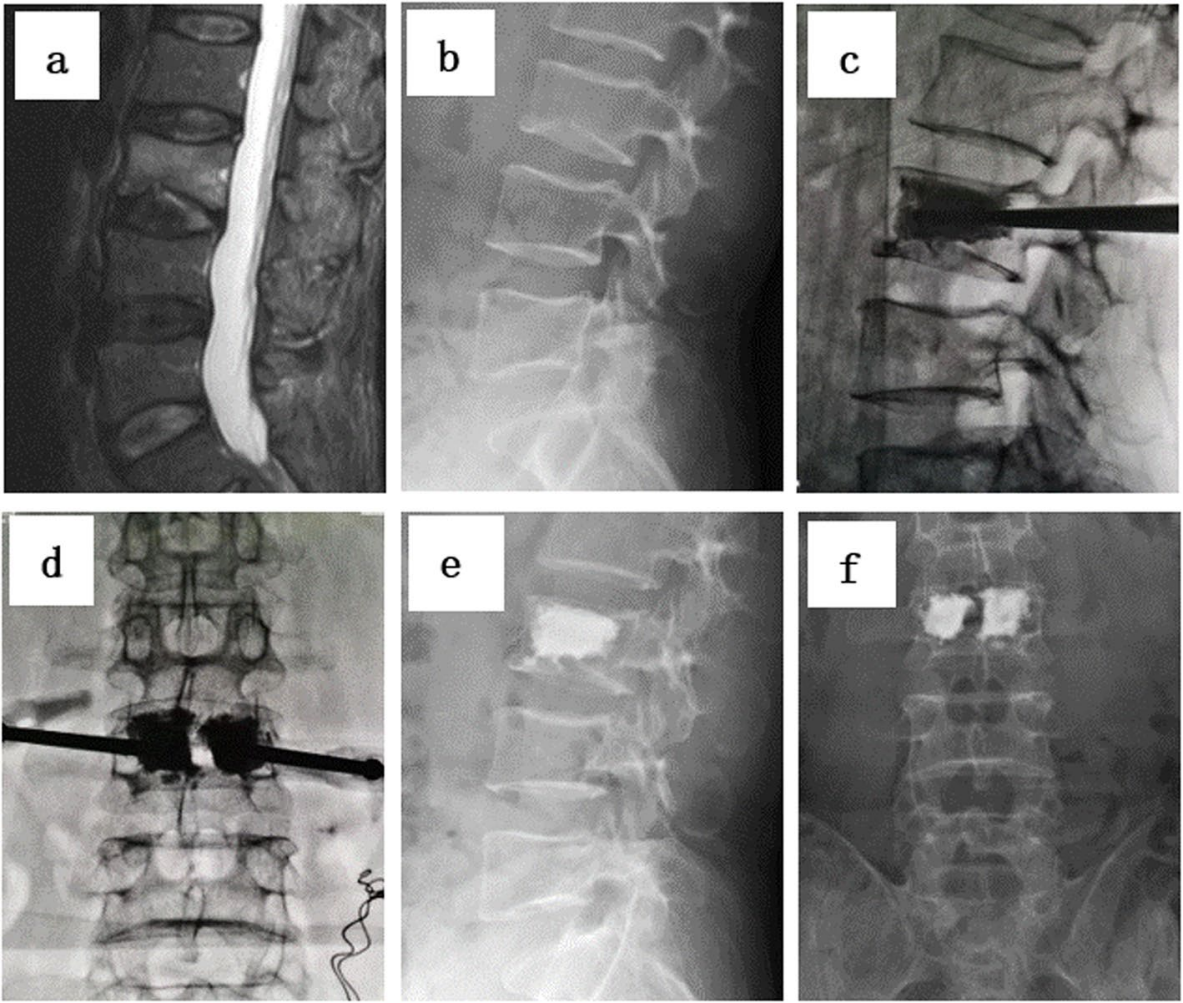
**a**-**f **Percutaneous kyphoplasty for the treatment of a L3 vertebral compression fracture in bipedicular approach. **a**-**b** Preoperative lateral radiograph and MRI showing a L3 vertebral compression fracture. **c**-**d** intraoperative view (**e**-**f**) Postoperative lateral radiograph showing cement distribution after undergoing PKP surgery of L3 vertebral compression fracture

**Fig. 2:**
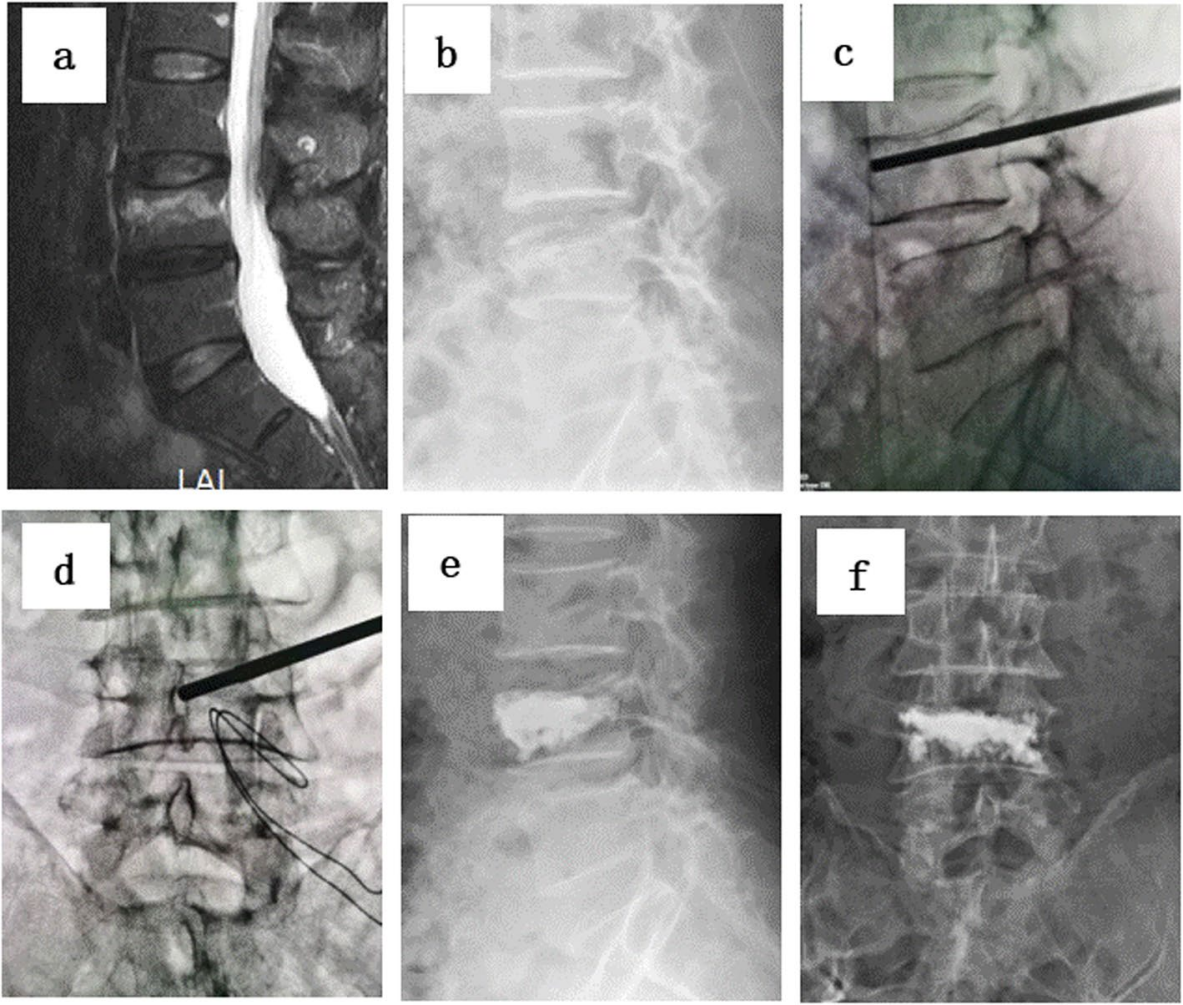
**a**-**f **Percutaneous kyphoplasty for the treatment of a L4 vertebral compression fracture in unipedicular approach. **a**-**b** Preoperative lateral radiograph and MRI showing a L4 vertebral compression fracture. **c**-**d** intraoperative view (**e**-**f**) Postoperative lateral radiograph showing cement distribution after undergoing PKP surgery of L4 vertebral compression fracture

A 1-cm skin incision was made percutaneously lateral to the desired entry point of the pedicle. A trocar (Shandong Guanlong Medical Utensils Co., Ltd., Jinan City, Shandong Province, China) in a cannula was inserted into the pedicle at the fractured vertebra using a pedicular approach as the working channel. After the trocar was removed, a balloon was placed in the working channel and slowly inflated to create a low-pressure cavity for cement injection. Inflation continued until the balloon pressure reached 300 psi. The balloon was then deflated and removed. Next, the PMMA cement was injected into the defect of the fractured body through the cannula under continuous fluoroscopic monitoring. The PMMA insertion was considered complete when it reached the posterior third of the vertebral body or had a tendency toward cortical, epidural, or anterior venous cement leakage. In the bipedicular approach, the same surgical steps are performed for both pedicles. In the unipedicular approach, the puncture angle was increased, with the end of the cannula placed as close to the midline as possible so that the inflated balloon could exceed the midline of the vertebra on the anteroposterior view to allow the PMMA to spread as much as possible throughout the vertebral body (Figs. 1 and 2).

### Measurements

**Fig. 3:**
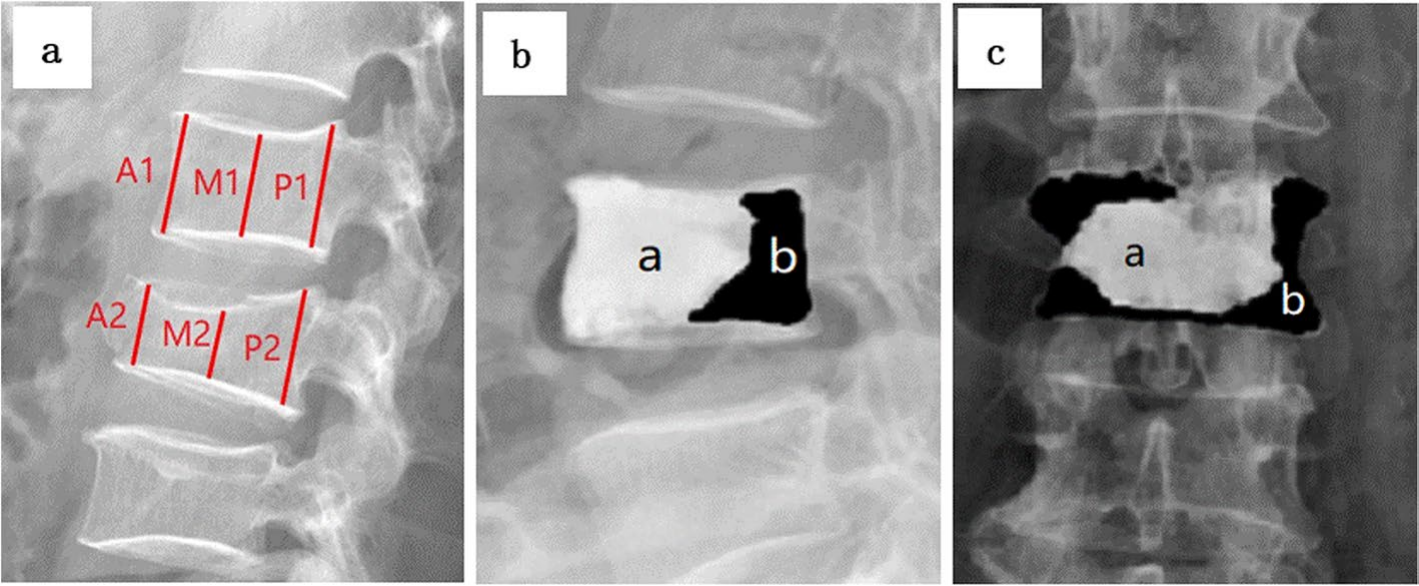
**a**-**c** Methods of measurements on images. **a** Lateral radiograph showing evaluation of the anterior vertebral body height ratio (AVBHr), middle vertebral body height ratio (MVBHr), and posterior vertebral body height ratio (PVBHr). AVBHr=A2/A1, MVBHr=M2/M1, and PVBHr=P2/P1. **b**-**c** Lateral and Frontal radiograph showing the evaluation of cement distribution. Cement distribution =a/(a+b)

Imaging findings and clinical outcomes were analyzed before and after surgery. Anterior, middle, and posterior vertebral body height ratios (AVBHr, MVBHr, and PVBHr, respectively) were measured using lateral radiographs, as described previously [11]. The methods used to measure the vertebral body height ratios are shown in Fig. 3. Vertebral height was defined as the endplate-to-endplate distance measured from the anterior aspect of the vertebral body on the lateral radiographs. Operation time, estimated blood loss, length of hospital stay, and complications were recorded. The visual analog scale (VAS) was used to evaluate analgesic efficacy (on a scale of 0–10), while the Oswestry Low Back Pain Disability Index (ODI) was used for functional assessment. Cement distribution was calculated using anteroposterior and lateral radiographs. Cement leakage was determined using X-ray films. The immediate and 2-year postoperative follow-up findings of all patients were recorded. Incidence of cement leakage from the vertebral body on postoperative radiographs and type of bone cement leakage were also recorded.

Furthermore, two independent spine surgeons performed the clinical evaluation of the patients. Additionally, three other spine surgeons assessed the radiographs.

### Statistical analysis

Statistical analyses were performed using the PASW Statistics for Windows (version 18.0; SPSS Inc., Chicago, IL, USA). Numeric variables are presented as means ± standard deviation. Repeated-measures analysis of variance (ANOVA) was used to analyze the differences between preoperative and postoperative VAS scores, ODI scores, and vertebral height restoration. Student’s t-test was used to compare measurements between the two groups. Nominal variables (sex, distribution of the fractured vertebra, and cement leakage) were presented as numbers (percentages) and compared using chi-square tests. *P* values < 0.05 were considered to indicate statistically significant differences.

## Results

The clinical characteristics of the 160 patients are summarized in Table 1. The mean ages of the patients in the unipedicular and bipedicular groups were 75.86 ± 7.74 years and 74.23 ± 8.17 years, respectively. Age, sex, injury time, BMI, segmental distribution, and morphological classification of fractures before surgery did not differ significantly between the groups. Operation time and blood loss were significantly reduced in the unipedicular group compared to the bipedicular group (32.01 ± 4.48 vs. 42.42 ± 6.01, 5.46 ± 1.22 vs. 8.73 ± 1.20; *p* < 0.05). There were no significant differences in the lengths of hospital stay between the two groups. Details are presented in Table 2.

**Table 1:** Baseline patient characteristics

	**Unipedicular group**	**Bipedicular group**	**t(**χ^2^**)**	**P**
NO of cases	82	78		-
Age	75.86 ± 7.74	74.23 ± 8.17	t = 1.300	0.196
Male/Female	9/73	15/63	*χ*^*2*^ = 2.137	0.185
BMI	23.77 ± 3.78	23.49 ± 3.57	t = 0.488	0.312
Distribution			*χ*^*2*^ = 0.267	0.875
L3	34	32		-
L4	31	32		-
L5	17	14		-
Vertebral morphometry			*χ*^*2*^ = 0.188	0.888
A (wedging)	25	26		
B (biconcavity)	47	44		
C (crush)	10	8		
Injure time(day)	4.53 + 3.52	4.20 + 3.89	t = 0.565	0.573

Data are presented as mean ± standard deviation

**Table 2:** Patient’s perioperative parameters comparison between unipedicular group and bipedicular group in this study

	Unipedicular group	Bipedicular group	*t(χ*^*2*^*)*	*P*
Operation time (min)	32.01 ± 4.48	42.42 ± 6.01	t = -12.465	< 0.001
Blood lose (mL)	5.46 ± 1.22	8.73 ± 1.20	t = -17.058	< 0.001
Hospital stay (days)	4.90 ± 1.46	4.79 ± 1.35	t = 0.482	0.630
Bone cement leakage	19(23.1%)	20(25.6%)	*χ*^*2*^ = 0.132	0.716
vein leakage	6	5	*χ*^*2*^ = 0.208	0.648
Intervertebral leakage	5	4	*χ*^*2*^ = 0.219	0.640
Paravertebral leakage	8	10	*χ*^*2*^ = 0.244	0.621

Data are presented as mean ± standard deviation

### Clinical outcomes

Most patients in both groups experienced excellent pain relief and improved physical abilities. The VAS scores decreased from 7.30 ± 1.01 preoperatively to 2.39 ± 0.76 postoperatively in the unipedicular group (*p* < 0.05) and from 7.23 ± 0.88 preoperatively to 2.38 ± 0.72 postoperatively in the bipedicular group (*p* < 0.05). The ODI scores decreased from 70.59 ± 4.22 preoperatively to 26.74 ± 2.50 postoperatively in the unipedicular group (*p* < 0.05) and from 70.88 ± 3.66 preoperatively to 26.58 ± 2.46 postoperatively in the bipedicular group (*p* < 0.05). The VAS and ODI scores did not differ significantly between the two groups either postoperatively or 2 years postoperatively. The operation time and blood loss in the unipedicular group were less than those in the bipedicular group (32.01 ± 4.48 vs. 42.42 ± 6.01, 5.46 ± 1.22 vs. 8.73 ± 1.20; *p* < 0.05). The details are presented in Tables 2 and 3.

**Table 3:** Clinical comparisons unipedicular group and bipedicular group in this study

	Unipedicular group	Bipedicular group	*t*	*P*
VAS				
Preoperative	7.30 ± 1.01	7.23 ± 0.88	0.492	0.623
Postoperative	2.39 ± 0.76*	2.38 ± 0.72*	0.048	0.962
2 years Postoperative	1.75 ± 0.62*	1.68 ± 0.63*	0.773	0.441
ODI				
Preoperative	70.59 ± 4.22	70.88 ± 3.66	-0.458	0.647
Postoperative	26.74 ± 2.50*	26.58 ± 2.46*	0.424	0.672
2 years Postoperative	16.77 ± 1.71*	16.72 ± 1.70*	0.190	0.850

Data are presented as the mean ± standard deviation. ODI:Oswestry disability index; VAS:visual analogue scale. * Repeated-measures analysis of variance was used for statistical analysis. There were significant differences (*p* < 0.05) between the postoperative or final 2 years postoperative and preoperative values in these two groups

### Radiographic evaluation

The anterior, middle, and posterior vertebral height ratios improved from (85.46% ± 10.26%, 80.32% ± 13.81%, and 90.87% ± 8.91%, respectively) preoperatively to (92.34% ± 9.42%, 86.58% ± 14.22%, and 92.50% ± 8.86%, respectively) postoperatively in the unipedicular group and from (85.69% ± 11.22%, 77.26% ± 12.81%, and 88.81% ± 9.96%, respectively) preoperatively to (90.86% ± 8.98%, 85.26% ± 11.84%, and 90.03% ± 8.39%, respectively) postoperatively in the bipedicular group. The cement distribution on the lateral radiographs did not differ significantly between the two groups (62.17% ± 11.40% vs. 62.05% ± 10.26%; *p* > 0.05). However, the anteroposterior radiographs showed a higher cement distribution in the bipedicular group compared to that in the unipedicular group (57.78% ± 11.36% vs. 52.13% ± 10.39%; *p* < 0.05). Bone cement exceeded the midline of the vertebral body in all patients. The radiographic results are presented in Table 4.

**Table 4:** Radiological comparisons unipedicular group and bipedicular group in this study

	Unipedicular group	Bipedicular group	*t*	*P*
AVBHR(%)				
Preoperative	85.46 ± 10.26	85.69 ± 11.22	-0.137	0.892
Postoperative	92.34 ± 9.42*	90.86 ± 8.98*	1.018	0.310
2 years Postoperative	88.63 ± 10.08*	86.35 ± 8.78*	1.531	0.128
MVBHR(%)				
Preoperative	80.32 ± 13.81	77.26 ± 12.81	1.448	0.150
Postoperative	86.58 ± 14.22*	85.26 ± 11.84*	0.636	0.526
2 years Postoperative	84.59 ± 14.34*	82.55 ± 11.27*	0.996	0.321
PVBHR(%)				
Preoperative	90.87 ± 8.91	88.81 ± 9.96	1.628	0.105
Postoperative	92.50 ± 8.86*	90.03 ± 8.39*	1.814	0.072
2 years Postoperative	90.27 ± 9.47*	88.55 ± 8.81*	1.184	0.238
Cement distribution(%)				
Anteroposterior radiographs	52.13 ± 10.39	57.78 ± 11.36	-3.283	< 0.001
lateral radiographs	62.17 ± 11.40	62.05 ± 10.26	0.070	0.944

Data are presented as the mean ± standard deviation. AVBHR: anterior vertebral body height ratio; MVBHR: middle vertebral body height ratio; PVBHR: posterior vertebral body height ratio

^*^ Repeated-measures analysis of variance was used for statistical analysis. There were significant differences (*p* < 0.05) between the postoperative or the 2 years postoperative and preoperative values in these two groups

### Complications

Leakage of bone cement outside the vertebral body was observed in both groups on the postoperative anteroposterior and lateral radiographs. The bone cement leakage rates were 23.1% (19 of 82) in the unipedicular group (six in the disc, five in the vertebral vein, and eight in the paravertebral vein) and 25.6% (20 of 78) in the bipedicular group (five in the disc, four in the vertebral vein, and 10 in the paravertebral vein) and did not differ significantly between the two groups (*p* = 0.716) (Table 2). None of the bone cement leakages required special treatment and no clinical symptoms were attributed to cement leakage. No other major postoperative complications, such as neurological damage, hemorrhage, infection, or pulmonary embolism, were observed.

## Discussion

Both unilateral and bilateral PKP have proven effective in the treatment of OVCFs. Previous studies have shown that both the unipedicular and bipedicular approaches can achieve considerable improvements in vertebral height restoration and pain relief. However, few studies have been conducted on the lower lumbar spine. While Rebolledo et al. [12] compared unilateral and bilateral PKP in the lower lumbar vertebrae, they included only seven patients. Chen et al. [13] reported four cases of L3 and L4 in 2010. The present study included 66 cases of L3, 63 cases of L4, and 31 cases of L5 OVCFs, respectively, which have not been previously reported.

### Clinical outcomes

The primary purpose of kyphoplasty is to relieve pain and improve patient function, thereby improving quality of life. In the present study, the VAS and ODI scores significantly improved in both groups postoperatively (*p* < 0.05), with no statistically significant difference between the unilateral and bilateral groups, a finding consistent with previous reports. Wang et al. [7] reported better VAS and ODI scores in the bipedicular group than in the unipedicular group postoperatively; however, the difference was not statistically significant. Zhang et al. [14] reported superior 3-month follow-up outcomes of the bipedicular approach for PKP compared to the unipedicular approach. Some articles reported contradictory findings; Song et al. [8] reported greater improvement in VAS scores in the unipedicular group than in the bipedicular group. However, most previous studies focused on the thoracolumbar spine, with few reports on the lower lumbar spine; thus, additional studies are required**.**

### Radiographic evaluation

Both techniques can effectively restore vertebral body height. In 2010, Chen et al. [15] reported that bipedicular PKP was more effective than unipedicular PKP in improving the vertebral height. More recently, in their 2020 study, Lee et al. [9] reported no differences in height restoration between the two techniques. We also concluded that both techniques provided similar restoration of vertebral height, with no differences in the anterior or middle vertebral heights post-operatively. However, the imaging findings in the present study showed an unremarkable degree of vertebral compression of the fractured segment in the lower lumbar region; therefore, the recovery of vertebral height postoperatively was limited in both groups.

Bone cement distribution is important for postoperative functional improvement and stabilization of the vertebral body, and likely plays a major role in pain relief [16–21]. Tan et al. [22] reported that a fully distributed bone cement can better restore the strength and maintain the height of the vertebral body. In the present study, the distribution of cement on the lateral radiographs did not differ between the two groups; however, the anteroposterior radiographs showed a wider distribution for the bipedicular approach than for the unipedicular approach. Owing to the large puncture angle, the end of the cannula should reach the midline as far as possible. Therefore, the cement exceeded the midline of the vertebral body in the anteroposterior radiographs of the selected cases in the unipedicular approach group. Chen et al. [17] showed that cement augmentation crossing the midline resulted in increased stiffness on both sides, with strong potential for achieving biomechanical balance. The present study showed similar radiographic and clinical outcomes between the unilateral and bilateral groups. Thus, unilateral kyphoplasty can provide stability for lower lumbar compression fractures when the cement exceeds the midline of the vertebral body.

### Complications

Kyphoplasty carries the risk of complications, including pulmonary embolism, cement leakage, neurological deficits, and even paraplegia [23, 24]. The most common complication of percutaneous vertebroplasty is cement leakage, particularly cortical and venous leakages. Bone cement can enter the pulmonary artery through the paravertebral vein and cause a pulmonary embolism, which can lead to death. Risk factors for postoperative cement leakage include cortical disruption, higher cement volume, intravertebral cleft, and solid cement distribution [25, 26]. Previous studies have demonstrated that the unipedicular approach results in fewer cement leaks. Zhang et al. [14] reported bone cement leakage rates of 20.8% (five of 24) in the unipedicular group and 34.6% (nine in 26) in the bipedicular group, which did not differ significantly between groups. Lee et al. [9] reported no significant differences in the leakage rates of cement into the intradiscal space (14% in the unipedicular group and 18% in the bipedicular group). A 2019 meta-analysis by Chen et al. concluded that the unilateral approach decreased the incidence of cement leakage in PKP [27]. Similar to previous reports, the cement leakage rate in the bipedicular group (25.6%) in the present study was higher than that in the unipedicular group (21.3%). However, there were no significant differences between the two groups.

### Limitations

The present study had some limitations. First, it was a retrospective analysis with incomplete data for some cases and inadequate follow-up time. Additionally, postoperative CT images were lacking, which may have more accurately reflected the distribution of bone cement postoperatively. Further prospective studies with longer or more frequent follow-ups are required to confirm our findings.

## Conclusions

The results of our study demonstrated that both unipedicular and bipedicular PKP techniques were effective for treating OVCFs in the lower lumbar region. However, the bipedicular approach provided better cement distribution than the unipedicular approach. We observed no statistically significant differences in pain relief, functional recovery, or vertebral height restoration between the two groups. However, the unipedicular approach has the advantages of short operation time, less blood loss, and less radiation exposure. In conclusion, the unipedicular approach may be clinically preferred because of its advantages.

## Data Availability

The datasets used and/or analyzed during the current study are available from the corresponding author on reasonable request.

## Abbreviations

ANOVA: Analysis of variance
BMI: Body mass index
MRI: Magnetic resonance imaging
ODI: Oswestry Low Back Pain Disability Index
OVCFs: Osteoporotic vertebral compression fractures
PKP: Percutaneous balloon kyphoplasty
PMMA: Polymethyl methacrylate
STIR: Short tau inversion recovery
VAS: Visual analog scale
